# Novel Information-Driven Smoothing Spline Linearization Method for High-Precision Displacement Sensors Based on Information Criterions

**DOI:** 10.3390/s23229268

**Published:** 2023-11-18

**Authors:** Wen-Hao Zhang, Lin Dai, Wang Chen, Anyu Sun, Wu-Le Zhu, Bing-Feng Ju

**Affiliations:** State Key Laboratory of Fluid Power & Mechatronic Systems, Zhejiang University, Hangzhou 310058, China; wenhao_zhang@zju.edu.cn (W.-H.Z.); dailin@zju.edu.cn (L.D.); chenwang_plus@zju.edu.cn (W.C.); anyusun@zju.edu.cn (A.S.); mbfju@zju.edu.cn (B.-F.J.)

**Keywords:** sensor, linearization, information criterion, model selection, smoothing spline

## Abstract

A noise-resistant linearization model that reveals the true nonlinearity of the sensor is essential for retrieving accurate physical displacement from the signals captured by sensing electronics. In this paper, we propose a novel information-driven smoothing spline linearization method, which innovatively integrates one new and three standard information criterions into a smoothing spline for the high-precision displacement sensors’ linearization. Using theoretical analysis and Monte Carlo simulation, the proposed linearization method is demonstrated to outperform traditional polynomial and spline linearization methods for high-precision displacement sensors with a low noise to range ratio in the 10^−5^ level. Validation experiments were carried out on two different types of displacement sensors to benchmark the performance of the proposed method compared to the polynomial models and the the non-smoothing cubic spline. The results show that the proposed method with the new modified Akaike Information Criterion stands out compared to the other linearization methods and can improve the residual nonlinearity by over 50% compared to the standard polynomial model. After being linearized via the proposed method, the residual nonlinearities reach as low as ±0.0311% F.S. (Full Scale of Range), for the 1.5 mm range chromatic confocal displacement sensor, and ±0.0047% F.S., for the 100 mm range laser triangulation displacement sensor.

## 1. Introduction

### 1.1. Background and Importance of Sensor Linearization

Sensors are the fundamental components in modern mechatronic systems, which typically transform a physical/chemical/biological variable into an interpretable signal for human or electronic processors. The general information flow of sensors could be summarized into an information encoding–decoding process: input physical values are encoded into electronic or mechanical signals and then decoded by signal processing modules to output the measurement value. According to the types of their input and output values, sensors can be categorized into different types such as displacement sensors, force sensors, pressure sensors, strain sensors, temperature sensors, etc. [1]. While their working principles may differ, these sensors share a common goal of achieving high measurement accuracy by reconstructing the detected quantities as close as possible to the true physical values. The nonlinearity, noise, and drift are the three fundamental error sources affecting the final performance [2]. Unlike noise and drift, which are random errors mainly generated in the signal capture stage, the nonlinearities are repeatable systematic errors that must be measured, using a more accurate instrument, and properly dealt with in the processing stage [3]. This process is called linearization. Relevant research about sensor linearization could be found for flow sensors [4], angle sensors [5], pressure sensors [6], displacement sensors [7], and even soft sensors [8]. In general, the linearization process must be carried out for most type of sensors before they can achieve high precision [9].

Among the above-mentioned sensors, displacement sensors are a typical type of physical sensor which measure the change in length unit and cover wide measurement applications such as position, motion, profile, vibration, and deformation [10]. Typical displacement measurement sensors include non-contacting types like laser displacement sensors [11,12], chromatic confocal sensosr [13], laser interferometers [14], and capacitance sensors [15], as well as contacting types like contacting probes [16] and linear variable differential transformers [17]. For displacement sensors, the purpose of linearization is to retrieve accurate physical displacement from signals detected by sensing electronics such as voltage, current, and signal peak position. The linearization could be divided into a three-step process:

(1) Nonlinearity measurement: measure the discrete nonlinearity deviations of the sensor using a traceable instrument with higher accuracy;
(2) Nonlinearity reconstruction: construct an accurate mathematical model of nonlinearity from the discrete measurement data;
(3) Nonlinearity correction: implement a real-time or off-line correction of the new measurement results using the constructed linearization model.

### 1.2. Review of Current Research on Sensor Linearization

A comprehensive review for the implementation of calibration systems for high-precision displacement sensors could be found in the literature [18]. Currently, most of the attention has been paid to the design of high-precision nonlinearity measurement systems to improve the nonlinearity measurement accuracy [19,20,21,22,23] and the development of high-performance software algorithms in processing systems like microcontrollers to correct the nonlinearity more efficiently [24,25,26].

For nonlinearity measurements, Seppä et al. established a novel linearization system that can correct sub-fringe periodic nonlinearity of heterodyne laser interferometers via a capacitive sensor and achieved picometer-level repeatability [19]; Giusca et al. adopted five step height artefacts ranging from 19 nm to 17 µm to calibrate the z-axis nonlinearity of aerial measuring instruments. By using a linear regression model, they achieved a nonlinearity of ±0.043% F.S. for a coherence scanning interferometer, ±0.065% F.S. for an imaging confocal microscope, and ±1.60% F.S. for a contact stylus instrument [20]; Brand and Herrmann developed a laser interferometry system to linearize a 13 µm range inductive displacement sensor and achieved a low theoretical fitting residual of ±0.012% F.S. [21]; Köning et al. investigated the influence of periodic interferometer errors on the calibration of capacitance displacement sensors, with a special focus on the oscillation of sensor sensitivity introduced by polarization mixing during linearization [22]; Haitjema et al. created a traceable calibration system for nanometer sensors based on a Fabry–Perot interferometer and achieved picometer resolution and nanometer uncertainty in a 300 µm range [23]. In a calibration experiment with this system, the nonlinearity of a capacitive displacement sensor was measured to be ±2% F.S. in a 100 µm subrange.

For the implementation of nonlinearity correction algorithms, Erdem compared the computational performance of six linearization algorithms on microcontrollers with a nonlinear optical displacement sensor and offered insights into the selection and implementation of an optimal linearization algorithm [24]; Bouhedda proposed a neuro-fuzzy architecture for linearization on FPGA (Field Programmable Gate Array) and reduced the required computational resource by over 40% compared to traditional computation methods [25]; Sonowal implemented four linearization algorithms, including piecewise linearization, lookup table, interpolation, and artificial neural networks, on FPGA and conducted a thorough comparison of their accuracy, speed of operation, and cost of implementation [26].

The aforementioned works have covered the first and third steps for linearization. However, the second step regarding nonlinearity reconstruction has remained insufficiently explored in terms of how to construct a linearization model that best reveals the nonlinear characteristics of the displacement sensors from small amounts of nonlinearity measurement data while also adequately addressing the noise. Conventionally, polynomial models (PL) are adopted as standard methods for linearization [9,27,28,29]. For example, Mao et al. employed a third-order PL with a C2 continuity to linearize two separated intervals of interest for a laser displacement sensor, improving its measurement accuracy by up to 70% compared to linear interpolation [28]; Pereira et al. introduced an adaptive self-calibration method via progressive polynomial interpolation, which dynamically updates the model coefficients with new measurement results [27]. The polynomial linearization models can achieve an adequate linearization result for smooth nonlinearities. However, for high-precision displacement sensors with varying local nonlinearity change, polynomial linearization models are restrained by excessive residual error due to the oscillation at the edges called Runge’s Phenomenon [30].

Attempts have been made by researchers to use data-driven machine learning approaches like the piecewise linear model (PWL) [31,32,33,34] and artificial neural networks (ANN) [35,36,37] to model nonlinearity. The piecewise linear model is computationally efficient to calculate, but the remaining fitting error in each linear segment impairs the achievable accuracy, making it more suited to sensors with smooth nonlinearities. Hille et al. utilized a PWL model for the hardware linearization of on-board integrated smart sensors, achieving both low cost and high memory efficiency [31]; Gao et al. introduced a high level canonical PWL model with alterable lattice divisions, which reduced the necessary partitions of PWL models while maintaining satisfactory approximation precision, achieving an average of over 30% error reduction in linearizing a diesel hydrotreater [32]; Srinivasan et al. employed a PWL model constructed by the include angle method to determine the optimal segmentation of piecewise linear models, attaining a nonlinearity of ±0.12% F.S. for a J-type thermocouple and ±0.41% F.S. for a K-type thermocouple [33]; Marinov et al. developed a generalized PWL approach for linearizing sensors with strictly convex or concave nonlinearities, which can balance between the linearizing accuracy and computation efficiency for microcontroller implementations [34].

Apart from PWL, ANN methods have exhibited high potential in sensor linearization. Marques et al. explored the application of ANN in compensating the nonlinearities for arbitrary types of sensors and successfully reduced the nonlinearity error of a negative temperature coefficient resistor sensor from ±3.33% F.S. to ±0.42% F.S. over a 60 °C range [35]; Anandanatarajan et al. adopted both PL and ANN approaches for linearizing thermal sensors. By applying high order PL, they reduced the nonlinearity of a K-type thermocouple to ±1.02% F.S. and that of a thermistor to ±0.14% F.S. [29], which were further decreased to ±0.001% F.S. and ±0.065% F.S., respectively, using ANN approaches [36]; Abdin et al. assessed both circuit-based hardware linearization and ANN-based software linearization for a thermistor, demonstrating that software linearization with a simple 1-5-1 ANN architecture could achieve a wider linearization range than hardware approaches [37]. While neural networks have achieved good performance in certain applications, their practical application is challenged by limited data availability for training [38].

Advanced curve models with enhanced local controllability, such as Bezier curves [39,40] and Splines [41,42], have been employed for sensor linearization. Hua et al. [39] and Song et al. [40] utilized Bezier curves to develop linearization models for potentiometers in displacement measurements and demonstrated their superiority over PL methods via comparative experiments. By applying the Bezier curve linearization method to a potentiometer within a linear series elastic actuator, the method significantly reduced the displacement control error from 14.2 mm to 4.2 mm. In the application of splines, Wang et al. introduced a recursive S-spline model to enhance the computational efficiency for the on-chip training and linearization of smart sensors [41]. Their simulation experiments yielded a nonlinearity of ±0.005% F.S for an S-type thermocouple and ±0.175% F.S. for a NTC thermistor; Dong et al. employed an improved elastic clonal selection algorithm to construct an S-spline for the contour reconstruction of a pre-calibrated commercial laser displacement sensor’s measurements [42] at the application level, achieving a contouring error reduction from 282.91 µm to 252.55 µm in an API thread measurement experiment.

An overview of the literature related to sensor linearization is presented in Table 1. This overview highlights a predominant emphasis on designing models to improve the approximation residuals within the current research. However, a significant limitation of the current methods is the insufficient consideration of noise during linearization. The limited effectiveness of nonlinearity modeling methods has constrained further improvements in the measurement accuracy of high-precision displacement sensors. Consequently, there is a clear need for a linearization method that can adeptly handle both complex nonlinearities and noise [18,19,42].

### 1.3. Objective and Arrangement of This Paper

In this paper, we propose a novel information-driven linearization method by integrating smoothing spline with three standard information criterions and one newly devised criterion adapted for high-precision displacement sensors. The newly proposed method is different from conventional methods in that it can automatically select the best model parameter from the information in the data and the model itself, such as the number of data points, the residual sum of squares of fitting results, and most importantly, the freedom of the model. In this way, it can handle rough nonlinearity characteristics and can balance between underfitting and overfitting for noisy nonlinearity measurement data without human intervention. Verification and comparison experiments were carried out on two types of self-developed high-precision displacement sensors to validate the accuracy and repeatability of the proposed method. The results demonstrate that the smoothing spline linearization model constructed using the new modified Akaike information criterion stands out compared to that constructed from other information criterions and outperforms the standard polynomial model and the non-smoothing spline model in managing noise and local fluctuations.

The remainder of this paper are arranged as follows. Section 2 introduces the working principles of the polynomial linearization, spline linearization, and the smoothing spline linearization methods with four different information criterions. Section 3 lays out the workflow for nonlinearity measurement and the overall framework for conducting and validating the linearization methodologies. Section 4 presents the theoretical validation of the proposed methods via Monte-Carlo simulation and the conducted linearization experiments on two different types of sensors. Section 5 gives the results of the experiments and discusses the findings from the validation and test results. Section 6 summarizes and envisions potential improvements for displacement sensors in future work.

## 2. Principle

To compensate the systematic errors without overfitting the random noise, a good linearization model must be constructed to deal with the trade-off between goodness of fit and overfitting. Here, the theory foundations of three types of linearization methods are introduced: (1) the polynomial model, (2) the non-smoothing spline, and (3) the newly proposed smoothing spline linearization method constructed from the Bayesian information criterion, the Akaike information criterion, the corrected Akaike information criterion, and the new modified Akaike information criterion.

### 2.1. The Polynomial Linearization Model

Polynomial regressions are the standard method for linearization. Given input displacement sensor data *x* and interferometer data *y*, their generalized form could be expressed as:

(1) $$y=\sum_{j=0}^{k}\beta_{j}x^{j}+\varepsilon ,$$

where ε is the error component and $\beta_{j}$ are the coefficients. Under a specified order *k*, a group of $\beta_{0}-\beta_{k}$ could be calculated according to the standard ordinary least square method. Therefore, to find the best fit polynomial is to search for the polynomial order under which the polynomial model achieves the smallest root mean squared error (RMSE) on nonlinearity measurements. The RMSE is a standard metric for evaluating the deviation of the results predicted via the constructed model compared to the actual physical measurements, given by:

(2) $$\mathrm{RMSE}=\sqrt{\frac{\sum_{i=1}^{n}{\left[y\left(x_{i}\right)-y_{i}\;\right]}^{2}}{n}}.$$

The traditional polynomial model is powerful in generating a moderate fitting model with few coefficients [43,44,45], but they suffer from drawbacks of decreased linearization accuracy due to oscillation when a high order is demanded for modelling sharp local nonlinearity undulations. As a global fitting model, the polynomial model involves all of the input data for fitting every coefficient. As a result, when a high order polynomial is required to deal with high local undulation, any small change in the local region can affect the entire model, resulting in increased sensitivity to the data and decreased stability in fitting. This limitation poses a significant challenge for polynomial models to accurately capture local variations in the data and results in reduced robustness when handling local undulation and noise in the input data. In such conditions, Spline models are superior for linearization, as they are piecewise, smooth, and well suited to dealing with local undulation.

### 2.2. The Non-Smoothing Spline Linearization Model (SP-FEDF)

Spline is a smooth curve controlled by a series of knots and is smoothly conjugated by piecewise polynomials [46]. When using a spline, the model passes through all the input points, with each point serving as a knot of the spline. The degree of freedom for fitting equals the number of points, resulting in an accurate fit that captures the local features of the data. Here, we use the notation SP-FEDF (spline with full equivalent degree of freedom) to denote the non-smoothing spline. The basic form of spline is the B–spline. Given n+1 knots, where $t_{0}<t_{1}<\cdots <t_{n}$, the S-spline is formed via the linear combination of *n* basis functions $b_{i,m}\left(x\right)$ of order *m*:

(3) $$M\left(t\right)=\;\sum_{i=1}^{n}P_{i}b_{i,m}\left(t\right),$$

where $b_{i,m}\left(x\right)$ is iteratively defined as Equation (4) from [47].

(4) $$\begin{array}{cc} b_{i,0}\left(x\right) & =\left\{\begin{array}{cc} 1, & x_{i}<x<x_{i+1}\\ 0, & otherwise \end{array}\right.\\ b_{i,m}\left(x\right) & =\frac{x-x_{i}}{x_{i+m}-x_{i}}b_{i,m-1}\left(x\right)+\frac{x_{i+m+1}-x}{x_{i+m+1}-x_{i+1}}b_{i+1,m-1}\left(x\right). \end{array}$$

Unlike the polynomial model, for which any local change would affect all the coefficients, spline is composed of a series of polynomial functions, with each part covering a certain range of the input data. This characteristic allows the spline model to fit the data with a much lower order than the polynomial model. The insensitivity to local undulation due to its piecewise structure makes spline a better choice for fitting local rough models than the polynomial. However, the “pass-all-data” characteristic of spline also makes it prone to overfitting the data, which can result in an overly complex model that is sensitive to noise. Consequently, improvements must be taken when using spline for linearization to ensure that the model accurately captures the underlying behavior of the data, while avoiding overfitting the noise.

### 2.3. The Proposed Information-Driven Smooth Spline Linearization Model

Smoothing splines are powerful tools for addressing the issue of oscillation, for polynomial models, and the issue of overfitting, for non-smoothing splines. The idea of smoothing spline is to allow for not passing through all the observed data points exactly to prevent overfitting. This can be realized by introducing the smoothing factor λ and the penalty term, composed of second-order derivatives to control the undulation of the curve and balance the goodness of fit against the measurement noise. The smoothing spline with *n* data points and order *m* can be expressed as Equation (5) with a modified loss function [48]. It adopts the product of a smoothing factor λ and the integral of the second derivative of the fitting function as the penalty term. However, determining the optimal smoothing factor for a given dataset can be a challenging task.

(5) $$S=\;\sum_{i=1}^{n}{\left[y_{i}-\;\sum_{j=1}^{m}P_{i}b_{i,m}\left(x_{i}\right)\right]}^{2}+\;\lambda \int_{x_{min}}^{x_{max}}{\left[\sum_{j=1}^{m}P_{i}{b_{i,m}}^{\prime \prime }\left(x\right)\right]}^{2}dx$$

Intrinsically, the smoothing operation is to use a spline with less knots than the number of points to prevent overfitting. The number of adopted knots is called the equivalent degree of freedom (EDF) [49], which is negatively correlated to λ. While λ is a continuous variable that requires discretization for optimizing, the EDF is intrinsically a discrete value with definitive physical interpretation that suits for optimizing [50]. Therefore, to construct a good smoothing spline linearization model is to find an EDF that achieves good predictions on new measurements. The fitting RMSE fails for this condition since it always decreases as EDF increases. Under extreme conditions, the selected smoothing spline becomes a cubic spline that passes through all the measured nonlinearity points and incorporates all the noise. Therefore, a valid parameter selection criterion is required to determine EDF for smoothing spline.

For smoothing splines, it is desired to find the best EDF that can predict the measurement results as close to the laser interferometers measurements as possible without over fitting the random noise of the measurement data. The key is to devise a suitable and concrete criterion for model selection [51]. To tackle this issue, the information-driven smoothing spline linearization method for sensors is proposed by incorporating three standard information criterions (the Bayesian information criterion, the Akaike information criterion, and the corrected Akaike information criterion) and one new information criterion (the modified Akaike information criterion) to enable the automatic determination of smoothing factors for linearization models. In statistical analysis for model selection problems, the Bayesian information criterion and the Akaike information criterion are the two mainstream metrics founded on information theory [52,53,54,55]. Variations of the Akaike information criterion are also significant techniques, of which the corrected Akaike criterion is the most important one [56]. To adapt to high-precision displacement sensors, a new modified Akaike information criterion with an alleviated penalty is proposed to further improve the performance of the selected model. The details of the smoothing spline linearization method combined with the corresponding four criterion strategies are introduced as follows.

**Criterion strategy 1**: The smoothing spline by the Bayesian Information Criterion (SP-BIC).

The Bayesian information criterion (BIC) is a pervasively adopted model selection metric in statistics derived by Gideon E. Schwartz in 1978 [57,58]. The basic idea of BIC is to add a penalty term to the maximum likelihood estimation to avoid overfitting. In model selection applications, a model with a lower BIC is preferred compared to one with a higher BIC. The standard expression of BIC is:

(6) $$\mathrm{BIC}=-2ln\;\left(\hat{L}\right)+\;k\;ln\;\left(n\right),$$

where *k* is the number of parameters of the model (in this case, the EDF), *n* is the number of unique sample points in the dataset, and $\hat{L}$ is the maximum likelihood estimation under specified *k*. For discrete measurements, the $\hat{L}$ is expressed as Equation (7).

(7) $$\hat{L}=\prod_{i=1}^{N}p(\theta_{i}|x_{i},\;y_{i})$$

In Equation (7), $\theta_{i}$ are the *k*-dimensional coefficients’ vector, $x_{i}$ are the values measured via the displacement sensor, and $y_{i}$ are the values measured via the reference interferometer. When the measurement noise conforms to the Gaussian distribution (validated by the experiments in this paper), the maximum likelihood estimation can be converted to the form of a residual sum of squares (RSS). To distinguish from the BIC information criterion, the notation SP-BIC is used to express the proposed smoothing spline sensor linearization model with EDF selected by BIC, which is formulated as:

(8) $$\mathrm{SP}\text{-}\mathrm{BIC}=\underset{\mathrm{EDF}\;\in \;[1,n]}{\arg\min}\left\{n\ln\left(\frac{\mathrm{RSS}}{n}\right)+\mathrm{EDF}\cdot \ln\left(n\right)\right\},$$

where

(9) $$\mathrm{RSS}=\;\sum_{i=1}^{n}{\left(y\left(x_{i}\right)-y_{i}\right)}^{2}.$$

**Criterion strategy 2**: The smoothing spline by the Akaike Information Criterion (SP-AIC).

The Akaike information criterion (AIC) was formulated by Hirotugu Akaike in 1974 [59] for statistical identification and selection of regression models. It estimates the relative information loss of candidate models based on information theory and selects the best model with minimal AIC among the candidates. Similar to BIC, the AIC is a function of both the likelihood estimation and the estimated model parameters *k* with different penalty terms:

(10) $$\mathrm{AIC}=-n\ln\left(\hat{L}\right)+2k.$$

For normally distributed errors, the proposed smoothing spline linearization model with EDF selected by AIC could be expressed as:

(11) $$\mathrm{SP}\text{-}\mathrm{AIC}=\underset{\mathrm{EDF}\;\in \;[1,n]}{\arg\min}\left\{n\ln\left(\frac{\mathrm{RSS}}{n}\right)+2\cdot \mathrm{EDF}\right\}.$$

**Criterion strategy 3**: The smoothing spline by the corrected Akaike Information Criterion (SP-AICc).

The corrected Akaike information criterion (AICc) is a variation of AIC to address the potential bias of AIC when the sample size is small. The AICc is the most widely adopted and most representative variation of the AICc criterion [56]. It is defined as:

(12) $$\mathrm{AICc}=\mathrm{AIC}+\frac{2k\cdot (k+1)}{n-k-1}.$$

The AICc criterion introduces a penalty function that is more stringent than the AIC, which favors models with fewer EDF for the same dataset. This criterion is particularly useful in applications like ecology engineering where the data are sparse and the noise is large, as it helps prevent the selection of overly complex models. However, for applications such as displacement sensors, the noise is significantly smaller compared to the input signal. In such a condition, the AICc might over-penalize the model, leading to suboptimal fitting results. This problem also exists for other similar information criterions like the Hannan–Quinn Information Criterion (HQIC) [56]. Here, we only adopt the most generally used AICc for comparison. For normally distributed errors, the proposed smoothing spline linearization model with EDF selected by AICc could be expressed as:

(13) $$\mathrm{SP}\text{-}\mathrm{AICc}=\underset{\mathrm{EDF}\;\in \;[1,n]}{\arg\min}\left\{n\ln\left(\frac{\mathrm{RSS}}{n}\right)+2\cdot \mathrm{EDF}+\frac{2\cdot \mathrm{EDF}\cdot (\mathrm{EDF}+1)}{n-\mathrm{EDF}-1}\right\}.$$

**Criterion strategy 4**: The smoothing spline by the new Modified Akaike Information Criterion (SP-MAIC).

For high-precision displacement sensors like chromatic confocal displacement sensors and laser triangulation displacement sensors, the relative ratio between the root mean square of its measurement noise and its measurement range is small and typically in the 10^−5^ range. The measurement data are therefore fine observations of the system’s nonlinearity characteristics. In contrast to applications like in ecology, these applications have a low noise–signal ratio. In such low-noise applications, it is possible for the BIC, AIC, and AICc metric to select an over-simplified model due to excessive penalty, thus restricting the improvement of linearization accuracy. More information from the measurement data should be incorporated into the linearization model to make better predictions compared to large noise datasets. Hereby, the adjustment coefficient is introduced to alleviate the penalty term in AIC, where the new modified Akaike information criterion (MAIC) is formulated by:

(14) $$\mathrm{MAIC}=-n\;ln\left(\hat{L}\right)+\frac{2k}{n-k}.$$

It is noteworthy that the aim of MAIC differs from that of the AICc criterion, as well as other derived criterions, in that while the AICc endeavors to construct simpler models to mitigate overfitting, the MAIC is specifically designed to construct a more intricate and accurate model that effectively accommodates the high-precision characteristics of displacement sensors. This is accomplished by adopting a more moderate penalty term. In Equation (14), when *k* is small, the penalty term coefficient 1/(n−k) is small, thus allowing the MAIC to decrease rapidly to the desired range. As *k* increases, the penalty term also increases quickly to exclude overfitted models. Note that *k* is always smaller than *n* because the coefficients of the linearization model would never exceed the number of measurement data for fitting.

Compared to BIC, AIC, and AICc, the MAIC metric allows for higher EDF to raise the information adopted for constructing the smoothing spline linearization model. For measurement data with normally distributed noise, the proposed smoothing spline linearization model in RSS form is expressed by:

(15) $$\mathrm{SP}\text{-}\mathrm{MAIC}=\underset{\mathrm{EDF}\;\in \;[1,n]}{\arg\min}\left\{n\ln\left(\frac{\mathrm{RSS}}{n}\right)+\frac{2\cdot \mathrm{EDF}}{n-\mathrm{EDF}}\right\}.$$

It is important to clarify that this paper does not propose the BIC, AIC, and AICc, which are widely-adopted standard criterions. Rather, this paper novelly integrates these information criterions into the construction of the smoothing spline model to optimize the smoothing parameter for sensor linearization, at the same time proposing the MAIC criterion with alleviated penalization to adapt to the linearization of high-precision displacement sensors with a typical 10^−5^ noise to range ratio.

## 3. Methodology

The preceding section establishes the theoretical foundation for constructing linearization models. A precise measurement of the nonlinearities of displacement sensors is essential to ensure an optimal linearization performance of these models. To achieve accurate and traceable measurement results, the displacement sensors must be calibrated using a traceable displacement measurement instrument with higher measurement accuracy. The laser interferometers are regarded as the standard traceable length reference [60]. They can achieve over 1 ppm nonlinearity (±0.00005%) to cover the nonlinearity of the displacement sensors, which is typically lower by two orders of magnitude.

**Figure 1:**
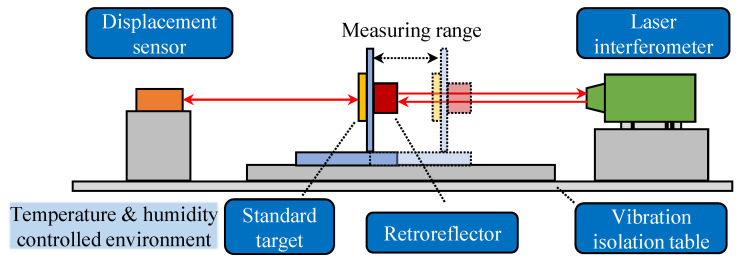
Scheme of the linearization system.

The basic principle of the designed linearization system is illustrated in Figure 1. The reference laser interferometer is mounted at the opposite direction of the displacement sensor. A standard target, usually an optical flat or high-precision ceramic plate, is placed between the displacement sensor and the reference laser interferometer on a servo motion stage. The measurement axis of the displacement sensor is aligned coaxial-wise with the measurement axis of the laser interferometer to minimize the Abbe error and the cosine error, which commonly result from the spatial offset and the angular deviation between the actual measurement axis and the desired axis to be measured. Since the laser interferometer is used as the length reference, the positioning error of the linear motion stage are excluded from the measurement process. To eliminate environmental errors, the whole system is seated on a vibration isolation stage in a temperature- and humidity-controlled environment.

For displacement sensors, the reference position is the zero-reading point after linearization and is defined by a reference distance starting from the front of the displacement sensor. After the standard target is moved to the reference position, the reading of the laser interferometer is set to zero to create an absolute calibration coordinate so the fitted linearization model would have a zero crossing at the point. The detailed workflow of the nonlinearity measurement system is arranged as follows:

Step 1: Fix the standard target to the motion table. Mount the displacement sensor and the laser interferometer to different sides of the workpiece. Move the target to the reference positions of the displacement sensor and create absolute calibration coordinates by setting the reading of the laser interferometer to zero;

Step 2: Move the motion table to near the end of the displacement measuring range. Record the data of the nonlinearized displacement sensor and the interferometer each time the standard target moves forward by a predefined calibration step;

Step 3: Feed the nonlinearity data to the linearization model and apply the model to the displacement sensor;

Step 4: Repeat the measurements. Record the data of both the displacement sensor and the interferometer each time the target moves by a predefined verification step;

Step 5: Evaluate the residual linearity errors by calculating the deviation between the displacement sensor reading and the laser interferometer reading.

**Figure 2:**
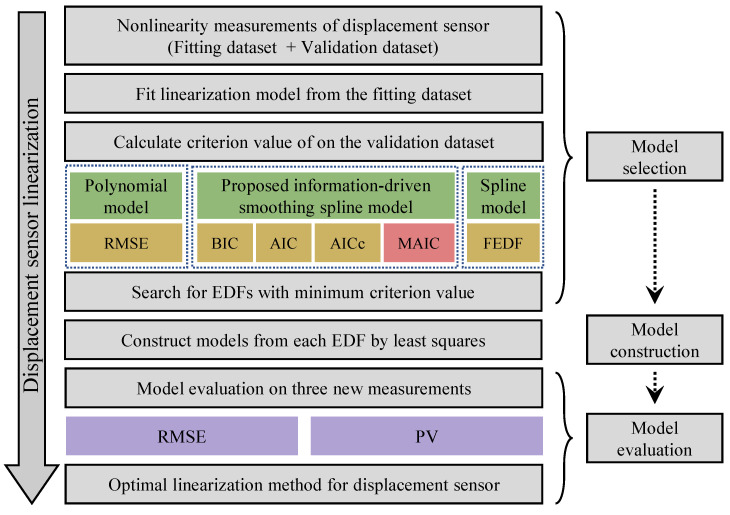
Workflow of the linearization model selection, construction, and evaluation process.

Note that the measured nonlinearity data in this process are physical observations of the true nonlinearity of displacement sensors coupled with random errors such as photon detective noise and electrical noise. After the nonlinearity data are measured along the sensing range, the linearization follows the cross-validation approach, as illustrated in Figure 2: (1) Model selection: two groups of data are measured for model fitting and validation, respectively, after which the optimal EDFs are determined by searching for the minimum criterion values calculated from the validation dataset; (2) Model construction: optimal linearization models under each linearization method are constructed from the selected EDFs via the least squares method; (3) Model evaluation: after the linearization model had been constructed, measurements can be carried out to test their performance on the new measurement datasets.

This systematic approach facilitates the linearization of displacement sensors and enables a detailed comparision of the performance of the linearization methods. A comparative analysis of the theoretical and practical aspects of these linearization methods is presented in the subsequent section.

## 4. Experiments

### 4.1. Theoretical Validation and Simulation

In this section, the theoretical performance of the four proposed information-driven linearization methods as well as the standard polynomial model and the non-smoothing spline model are evaluated from three key aspects: the local undulation of the targeted model, the noise level of the sampling dataset, and the number of samples in the measurement data. Among the three factors, the robustness to the local undulation and resistance to noise are critical metrics implying the reliability and adaptability of linearization methods.

#### 4.1.1. The Influence Factors for Sensor Linearization Methods

**Factor 1. The local undulation *A***: The local undulation refers to the local variability exhibited by a model function. Intuitively, the freedom of a model decides its complexity, which is positively correlated to its capability to handle local undulation. Compared to polynomial models, the information-driven smoothing splines are more effective to handle local undulation because they allow for higher freedom and can approximate the local undulations better via piecewise reconstruction.

**Factor 2. The noise level *N***: The noise-resistant capabilities of a linearization method is essential for achieving good linearization results. Ideally, a linearization model should demonstrate consistent high performance across different noise levels. This is key in physical applications since noise levels vary from one condition to another. For the proposed information-driven linearization methods, their superiority compared to the non-smoothing spline lie in the ability to adapt to the noise data, and thus, can achieve better linearization accuracy.

**Factor 3. The number of sampling points *P***: The number of sampling points could also influence the linearization results. As a general rule, more sampling points can furnish linearization models with more physical information to achieve better linearization results. Nevertheless, in practical conditions, the number of sampling points is commonly restricted to the hundreds, considering the time and effort required for measurement. Too many samples could also lead to overfitting. This trade-off must be balanced to avoid overfitting.

From the above analysis, it could be summarized in principle that the superiority of the proposed information-driven SP-MAIC lies in its ability to adopt a larger EDF to construct a more complex model (compared to the polynomial method and other smoothing spline methods), by using a milder penalty term, with the ability to deal with noisy data (compared to the non-smoothing spline method), by introducing the penalty term to avoid overfitting noise characteristics. To validate this, Monte Carlo simulations are conducted to compare the performance of the linearization methods in the following part of this section.

#### 4.1.2. Monte Carlo Simulation of Linearization Methods

Monte Carlo simulation is a known computational technique that involves using repeated random sampling to simulate the behavior of complex systems or processes [61]. To theoretically validate the performance of the proposed information-driven smoothing spline linearization model, nonlinear mathematical models are necessary for simulation. Without loss of generality, two typical nonlinear functions that commonly exist in physical system modeling are selected as the base function, including the cubic polynomial [62,63] and the sigmoid function [64,65,66]. To simulate the local undulation, a sine function is introduced into the two base functions, of which the amplitude serves as a quantitative measure of local undulation. Additionally, to simulate noise in nonlinearity data, Gaussian noise is added to the model, of which the standard deviation is used to quantify the noise level. The expression of the constructed cubic polynomial function (CPF) is:

(16) $$F_{c}\left(x\right)=1+x+x^{2}+x^{3}+A\cdot \sin\left(10\cdot x\right).$$

And the expression of the constructed sigmoid function (SF) is:

(17) $$F_{s}\left(x\right)=\frac{1000}{1+e^{-x}}+A\cdot \sin\left(10\cdot x\right).$$

**Figure 3:**
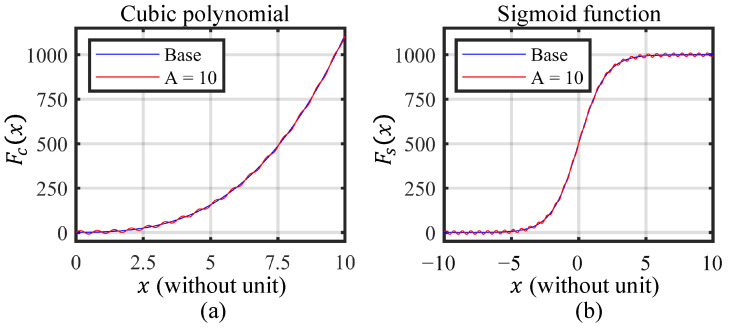
The two typical nonlinear functions for modeling physical systems. (**a**) The cubic polynomial function. (**b**) The sigmoid function.

For Equations (16) and (17), the input ranges for the cubic polynomial and the sigmoid function are set to [0, 10] and [−10, 10], respectively, for better visualization, as plotted in Figure 3. In particular, the base curves are the curves with zero local undulation (A = 0). The coefficients of the nonlinear functions are adjusted so that the maximum output amplitude is around 1000 in the valid range. This results in a noise–range ratio of approximately ±0.05% when a noise of value 1 is present. Additionally, The angular frequency of the sine component is set to 10 rad/unit, to make sure there are enough undulations across the input range.

In Figure 3, it shows the base functions’ standard for the two nonlinear models when A = 0. As A increases, the local undulation becomes more pronounced. The maximum undulation is set to 10 to cover possible undulation for the sensors. In the following parts, the notations A, N, and P were adopted for the local undulation, the noise level, and the number of samples, respectively. It should be noted in this simulation that (1) the noise level and local undulation are augmented in order to establish a more conspicuous differentiation between the diverse linearization methods and that (2) the input and output of the two nonlinear functions are dimensionless values, so the emphasis should be put on the relative comparison of linearization methods and the changing trend of the linearization results.

Based on the above settings, the Monte Carlo simulations are carried out according to the following steps:

Step 1. For a fixed local undulation *A* and a fixed sampling point *P*, *x* are evenly sampled from the input range of the nonlinear function;

Step 2. The theoretical output $\bar{y}$ is calculated from the sampled *x* via the nonlinear function;

Step 3. A Gaussian noise sequence with standard deviation *N* is generated and added to the theoretical output $\bar{y}$ to obtain the noisy output *y*, which, together with the sampled *x*, are used as nonlinearity measurements;

Step 4. The linearization methods in Section 2 are adopted to generate a linearization model from *x* and *y* and then estimate the output $\hat{y}$ from the constructed model.

Step 5. The performance of the linearization methods are evaluated by comparing the output of the linearization model $\hat{y}$ with the theoretical output *y* and calculating the root mean squared error (RMSE).

Step 6. Steps 3–5 are repeated a hundred times, each time with different random noise, to implement the Monte Carlo simulation. The average RMSE errors converge during the simulation and are calculated as the performance of the linearization model.

Step 7. To estimate the performance under different local undulation and sampling points, steps 2–6 are carried out after changing the local undulation *A* and the sampling points *P* as required.

Following the above simulation workflow, the conducted simulation and the simulated performance of the linearization methods are given as follows:

(1) Simulated Performance on the Local Undulation.

**Figure 4:**
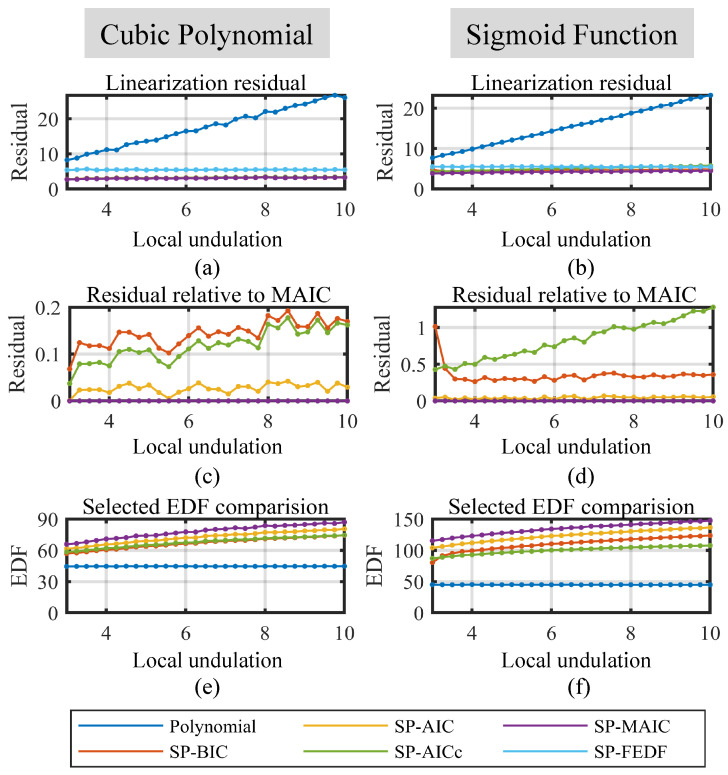
Performance of the linearization methods on different local undulation. (**a**) Linearization residuals on the CPF. (**b**) Linearization residuals on the SF. (**c**) Relative residuals to the SP-MAIC of the spline methods on the CPF. (**d**) Relative residuals to the SP-MAIC of the spline methods on the SF. (**e**) The best EDFs selected via smoothing spline methods at each local undulation for CPF. (**f**) The best EDFs selected via smoothing spline methods at each local undulation for SF.

To verify the performance of the linearization methods under different local undulation *A*, the number of sampling points and the noise level must be fixed to implement single-variable analysis. Without compromising generality, the number of sampling points is set to 200 as a representative case to sufficiently capture the nonlinearities of the function curves in Figure 3 while balancing the nonlinearity measurement duration for practical implementations. This choice also aligns with the number of points employed in the subsequent practical experiments. The noise level (the standard deviation, 1σ) is set to 1, resulting in a noise–range ratio of approximately ±0.05% F.S. for an amplitude of around 1000 in Figure 3. This value approximates the nonlinearity range observed in displacement sensors during the following practical experiments presented in Section 4.2. Note that under a standard deviation of 1, the 99.7% amplitude (3σ) of the noise is 3. So the minimum of the local undulation is set to 3 to make sure its amplitude exceeds 3σ of the noise. To test the performance of the linearization methods on different local undulation, the value is linearly sampled from 3 to 10 via a 0.25 step, which effectively constructs 29 sequential local undulations to analyze the variations in the linearization performance as the local undulation changes. The simulation results are plotted in Figure 4.

In Figure 4, the two columns correspond to the results for the cubic polynomial function and the sigmoid function, respectively. As shown in Figure 4a,b, the polynomial model exhibits notably higher residual error than the spline models within the valid range of local undulation. Specifically, the residual error of the polynomial model increases with local undulation, as it fails to effectively handle the local fluctuations of the functions. In contrast, the residuals of the spline linearization models remain relatively stable, indicating their robustness to different local undulation.

Among the spline models, the SP-FEDF model exhibits a higher residual error than the proposed information-driven smoothing spline models, which is the result of its overfitting of noise. Figure 4c,d presents the relative residual errors of the SP-BIC, SP-AIC, and SP-AICc compared to the SP-MAIC method, which are derived by substracting the residual error of the SP-MAIC for a more clear comparison. The positive relative residuals of other methods implies that the SP-MAIC model achieved a minimum residual error. Figure 4e,f further illustrates the reason—the EDF selected via the SP-MAIC model are higher EDF than other proposed information-driven spline models, allowing it to model local undulation better. Another notable trend is that the EDF selected by all four proposed smoothing spline models increases as the local undulation increases, since more rough characteristics require a more complex model to achieve a good compensation performance.

(2) Simulated Performance on the Noise Level.

**Figure 5:**
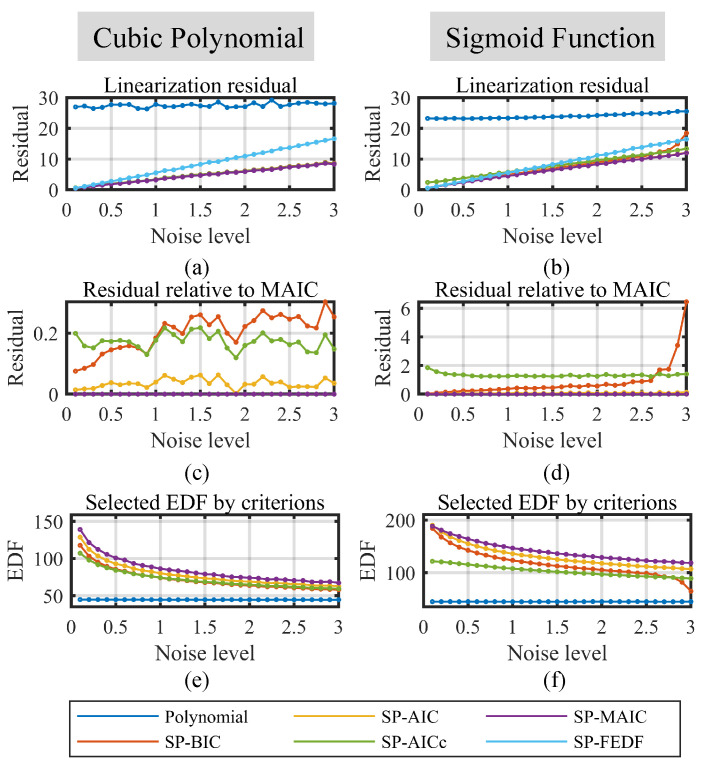
Performance of the linearization methods on different noise level. (**a**) Linearization residuals on the CPF. (**b**) Linearization residuals on the SF. (**c**) Relative residuals to the SP-MAIC of the spline methods on the CPF. (**d**) Relative residuals to the SP-MAIC of the spline methods on the SF. (**e**) The best EDFs selected by smoothing spline methods at each noise level for CPF. (**f**) The best EDFs selected by smoothing spline methods at each noise level for SF.

In this simulation, the number of samples is set to 200 in alignment with the choice in the preceding simulation and the local undulation is set to 10 to model highly varying local nonlinearity change, as illustrated in Figure 3, while the noise level varies from 0.25 to 3 via a 0.25 step to study the performance variations in the linearization methods as noise increases. The maximum of the noise level is set to 3 so its 3σ amplitude would be 9, which is still smaller than the amplitude of local undulation. The simulation results are plotted in Figure 5.

From Figure 5a,b, it can be seen that in the valid noise level range, the residual of the polynomial model are higher than the spline model. The residual of the polynomial model does not change much as the noise level changes because the oscillation of the polynomial model takes up a major part of the residual. The residual of the spline models rise as the noise level increases because of high variation from the noise. This contrast also demonstrates that the superiority of the spline models over the polynomial models becomes more apparent as the level of noise in the data decreases.

Among the smoothing spline models, SP-MAIC outperform the other three proposed information-driven models in general according to Figure 5c,d. In Figure 5d, the residual of SP-AIC is close to but still above the SP-MAIC. From Figure 5e,f, the EDFs selected by the information-driven spline models fall as the noise level rises. This is because smaller EDF is required to avoid constructing an overly complex model that overfits the noise. From the comparison, the EDF selected via the MAIC criterion is constantly the highest one, which conforms to the analysis in Section 2.3, where SP-MAIC has a more moderate penalty term.

(3) Simulated Performance on the Number of Samples

**Figure 6:**
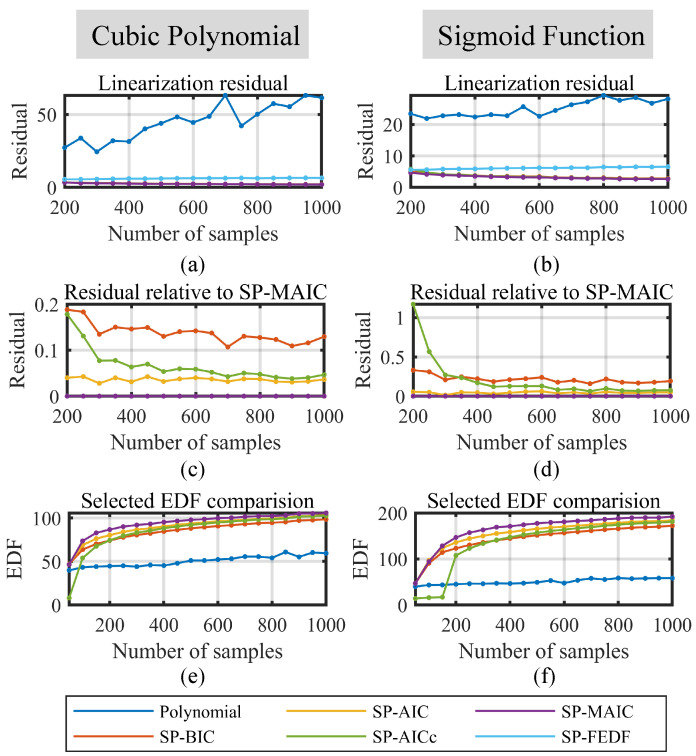
Performance of the linearization methods on different number of samples. (**a**) Linearization residuals on the CPF. (**b**) Linearization residuals on the SF. (**c**) Relative residuals to the SP-MAIC of the spline methods on the CPF. (**d**) Relative residuals to the SP-MAIC of the spline methods on the SF. (**e**) The best EDFs selected by smoothing spline methods at each number of samples for CPF. (**f**) The best EDFs selected by smoothing spline methods at each number of samples for SF.

A further simulation is conducted to verify the performance of the linearization methods with a different number of samples. In accordance with the parameter choices in the previous two simulations, the local undulation is set to 10 and the noise level is set to one. The results are plotted in Figure 6.

In this simulation, the range of the number of samples is strategically chosen from 200 to 1000 via 50-step high-precision displacement sensor linearization, where hundreds of samples are sufficient for capturing their nonlinearities while balancing the duration for the nonlinearity measurement process. While a larger number of samples typically improves the linearization outcomes, it also introduces potential drift due to the long measurement period. Assume that the measurement of each point requires five seconds, so a range of 200 to 1000 samples results in a duration of 16.7 to 83.3 min, which is reasonable for practical applications.

From Figure 6a,b, smoothing spline methods still perform better than the polynomial methods and SP-FEDF. However, the increasing trend of the polynomial linearization residuals contradicts the common idea where more samples should lead to better linearization results. This phenomenon results from the increased polynomial order in Figure 6e,f, which leads to an overly high-order polynomial model with worse performance. From Figure 6c,d, the SP-MAIC perform better than the other three information-driven spline linearization methods, and as the number of samples increases, their difference would converge to a stable level. In Figure 6e,f, the EDFs selected via the smoothing spline models increases as the number of samples increases, which agrees with the analysis in Section 4.1.1.

#### 4.1.3. Comparison of the Linearization Results

From the simulation results presented above, it can be summarized that compared to the polynomial and SP-FEDF models, the proposed information-driven smoothing spline linearization methods exhibit higher resilience to both noise and local undulation in principle. Among the smoothing spline linearization methods constructed from four different criterions, SP-MAIC demonstrates the best performance compared to others by allowing for a more complex model with a higher EDF. This conforms to the analysis conducted in Section 2.

The changing trends of the select EDF in the three simulations are also worth noting here. The EDFs selected by the proposed smoothing spline linearization methods with four criterions increase as the local undulation and numbers of sampling points increase, but decrease as the noise level increases. This further demonstrates the adaptability and versatility for the proposed methods—they can automatically adapt the constructed linearization model to the local undulation, the noise level, and the number of samples without human intervention, driven by information like the number of samples, the residual sum squared of fitting, and the freedom of the model from the input data and the constructed model only.

### 4.2. Verification Experiments of Linearization Methods

To verify the performance of the four proposed information-driven linearization methods, experiments were conducted on two types of self-developed displacement sensors including a chromatic confocal displacement sensor (CCDS), with a 1.5 mm range, and a laser triangulation displacement sensor (LTDS), with a 100 mm range. The LTDS and CCDS are chosen because they are both prevalent high-precision industrial applicable sensors with intrinsic optoelectronic nonlinearities that must be linearized before practical use, while they differ in terms of accuracy and range.

Two linearization calibration system were developed based on the scheme in Figure 1 to satisfy the high-precision requirements of the CCDS and the long-range requirement of the LTDS. In the nonlinearity measurement process, the fitting and validation dataset, each containing 200 measurement points, were generated from the calibration system. After linearization had been conducted, three groups of test datasets were adopted to evaluate the linearization performance. To maintain consistency with the measurement and validation datasets, the test datasets were also comprised of 200 data points.

#### 4.2.1. Verification Experiment on the Chromatic Confocal Displacement Sensor (CCDS)

**Figure 7:**
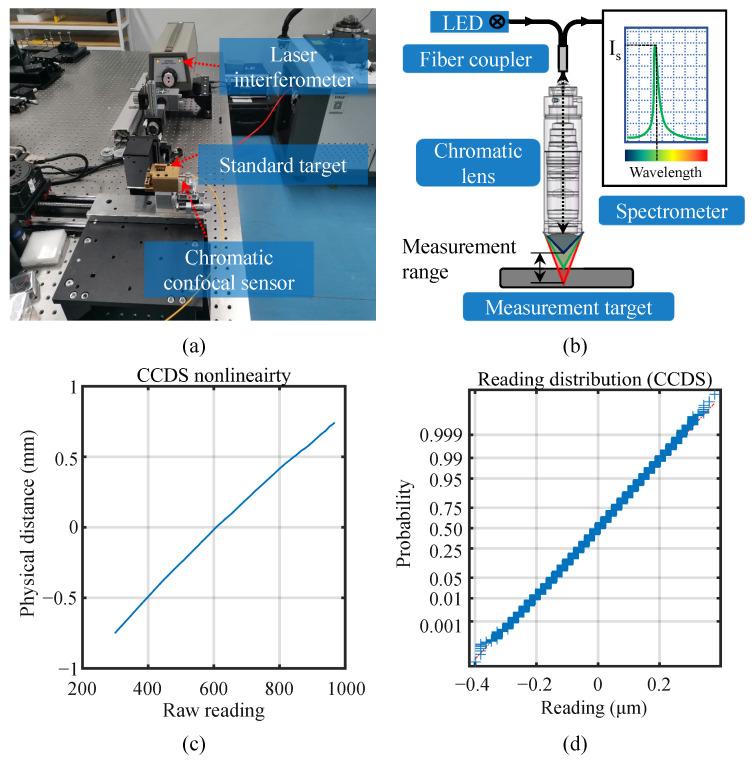
Linearization experiments on a self-developed CCDS. (**a**) Experimental setup. (**b**) Working principle of the CCDS. (**c**) The measured nonlinearity curve. (**d**) The distribution of measurement noise compare to Gaussian distribution.

In this experiment, the linearization methods were verified on the self-developed chromatic confocal sensor. Figure 7a shows the experimental setup. The nonlinearity of CCDS was measured via a NIST traceable Keysight E5530 laser interferometer (Keysight Technologies Inc., Santa Rosa, CA, USA). A linear motion stage driven by a five-phase stepper motor with submicron resolution carried the standard silver-coated plane reflective mirror to move along the range of CCDS for measuring nonlinearity. The working principle of self-developed CCDS is shown in Figure 7b. Visible white light emitted from an LED is transmitted into a chromatic dispersion lens group by an optical fiber to generate large chromatic dispersion along the measurement axis. When an object locates within the measuring range, the light focused on the surface of the object is reflected through the chromatic lens into a spectrometer and forms a peak on the photosensitive line CMOS sensor inside the spectrometer at the corresponding wavelength. Thus, the physical position of the measured object is uniquely mapped to the position of the peak on the CMOS sensor with nonlinearities from the spectrometer, the chromatic dispersion lens, and the processing electronics. The proposed linearization model would reconstruct this mapping relation and retrieve the physical position from the peak position detected via the CMOS sensor. The measured nonlinearity of CCDS is plotted in Figure 7c, where local undulations evidently exist.

Static measurement of noise was also conducted on the system to check the distribution of the sensor noise. After the standard plane mirror was moved into the sensing range and was kept static, the reading of CCDS was recorded at a 1 kHz rate for a 10 s period to obtain 10,000 static readings. The distribution probabilities of the readings, as shown in Figure 7d, conforms well to the standard normal distribution. So, the metrics from Equations (8), (11), (13), and (15) could be applied for model selection. The variance of the static measurement value was calculated to be 8.79 × 10^−5^ mm and the noise–range ratio is 5.86 × 10^−5^, considering the 1.5 mm measurement range of the CCDS.

In the model fitting stage, one group of measurement data was used for fitting and another was used for validating the fitted model. The same Gaussian noise as that in the static measurement was incorporated into the linearization model to check the performance of the fitting algorithm under noise.

**Figure 8:**
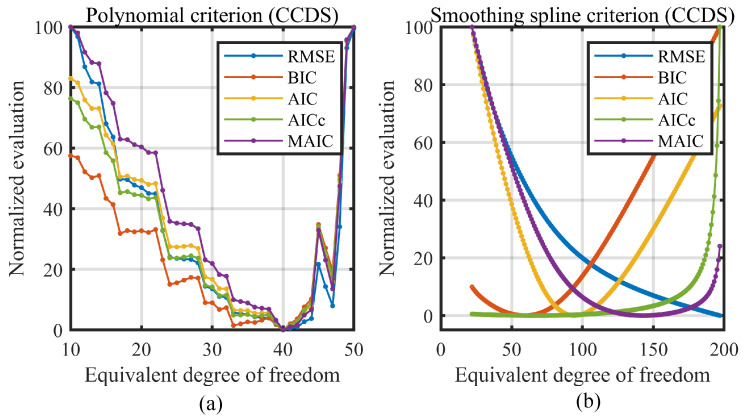
Change curve of model selection criterions on CCDS. (**a**) Criterions on the polynomial model. (**b**) Criterions on the smoothing spline model.

At each EDF, the fitted RMSE, BIC, AIC, AICc, and MAIC were calculated. Figure 8 plots the change curve of all the model selection criterions. Note that for the model selection process, only the relative value of the selection metric matters for finding which candidate model has the best performance under a certain selection criterion, so all the criterion values are linearly normalized to the [0, 100] range for comparing their changing trend.

Here, the degree of freedom for the horizontal axis in Figure 8a is the polynomial order plus one (for the intercept of the polynomial) and in Figure 8b, is the EDF for smoothing spline. For the polynomial model, although the RMSE, BIC, AIC, AICc, and MAIC are calculated and plotted in Figure 8a for reference, the RMSE criterion was adopted as the order selection metric as discussed in Section 2.1.

From Figure 8a,b, it can be found that the fitting RMSE for the polynomial model firstly falls and then rises as the order increases, but that for the smoothing spline model, decreases monotonically. This is identical to the discussion in Section 2.3. After the best EDF is determined using the information criterions, the smoothing spline linearization models were constructed via the chosen EDF and then tested on three measurements of the new data. The testing results are given in Section 5.

#### 4.2.2. Verification Experiment on the Laser Triangulation Displacement Sensor (LTDS)

**Figure 9:**
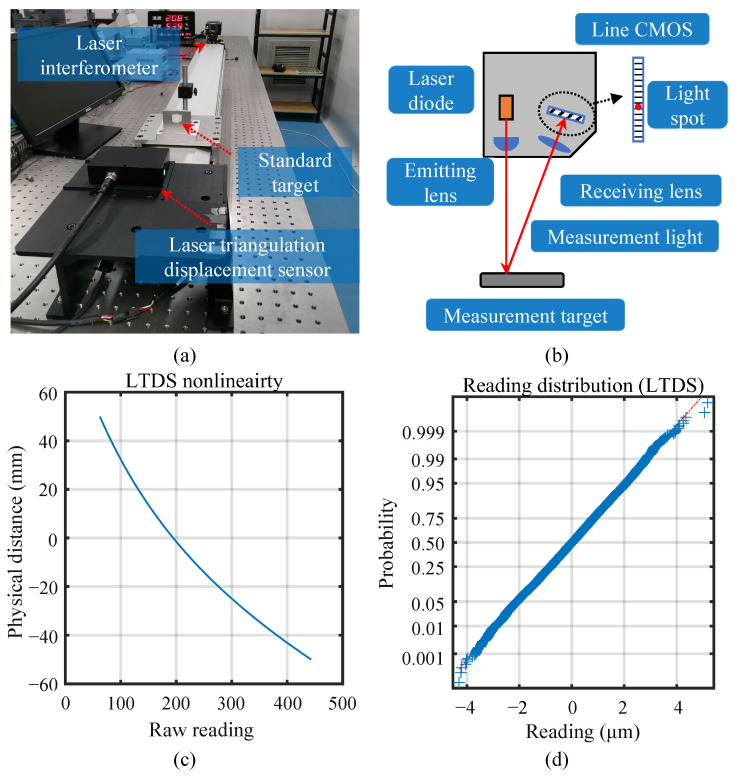
Linearization experiments on a self-developed LTDS. (**a**) Experimental setup. (**b**) Working principle of the LTDS. (**c**) The measured nonlinearity curve. (**d**) The distribution of measurement noise compare to Gaussian distribution.

To demonstrate the versatility of the proposed linearization methods on different displacement sensors, experiments were also carried out on a self-developed laser triangulation displacement sensor as shown in Figure 9a. Similar linearization system configuration and test procedures are used as those in Section 4.2.1, but with a different interferometer, standard target, and motion stage to demonstrate the versatility of the proposed linearization method.

In this setup, an API XD5 laser interferometer (Automated Precision Inc., Rockville, MD, USA) was adopted, which is also traceable to the SI length unit via NIST. A standard ceramic plate was used as the standard target, to guarantee good scattering ability, and a long-range linear stage driven by servo motors is adopted. Figure 9b illustrates the working principle of the LTDS. A 655 nm single-wavelength red laser emitted by the laser diode passes through the emitting lens and generates a light spot on the measurement target. The receiving lenses collect the light scattered from the target surface and focuses the returning light onto a line CMOS sensor. In this way, the position of the target is nonlinearly mapped to the position of the focused light spot on the CMOS sensor.

**Figure 10:**
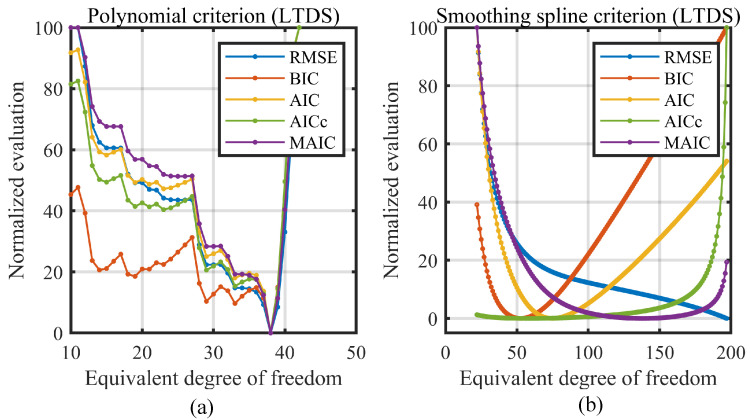
Change curve of model selection criterions on LTDS. (**a**) Criterions on the polynomial model. (**b**) Criterions on the smoothing spline model.

Figure 9c is the measured nonlinearity of LTDS, which is composed of the nonlinearities from the receiving lens, the CMOS sensor, and the processing electronics. The distribution of the sensing noise was also validated as shown in Figure 9d, which also conforms to the normal distribution. For the LTDS, the variance of noise was 1.243 × 10^−3^ mm and the variance–range ratio was 1.243 × 10^−5^. The change trend of evaluation criterions for polynomials and for smoothing spline was calculated and shown in Figure 10a and Figure 10b, respectively.

## 5. Results

Linearization results of the polynomial linearization methods, the proposed information-driven smoothing spline linearization methods with four different criterions (SP-BIC, SP-AIC, SP-AICc, and SP-MAIC), and the non-smoothing spline (SP-FEDF) are compared in this section on the two types of high-precision displacement sensors. Both the residual nonlinearity RMSE (indicating the dispersion of the residual error after linearization) and PV (peak-valley value, indicating the maximal possible measurement error of the sensor) are adopted to benchmark the performance of the linearization methods.

### 5.1. Linearization Results on the CCDS

Table 2 gives the EDF selected by each metric and their residual nonlinearity on the validation and test datasets. The proposed information-driven smoothing spline linearization method with four different criterions are distinguished from other methods in Table 2. Note here for polynomials that EDF is the order of the fitted polynomials, while for smoothing spline, it is the number of knots. The mean nonlinearities (Mean Nlt.) are the ratio between the averaged results of the three test groups and the full scale (F.S.) of the sensor range. This ratio summarizes the integrated performance of the linearization models and is used as the metric to compare the performance of different linearization methods.

**Figure 11:**
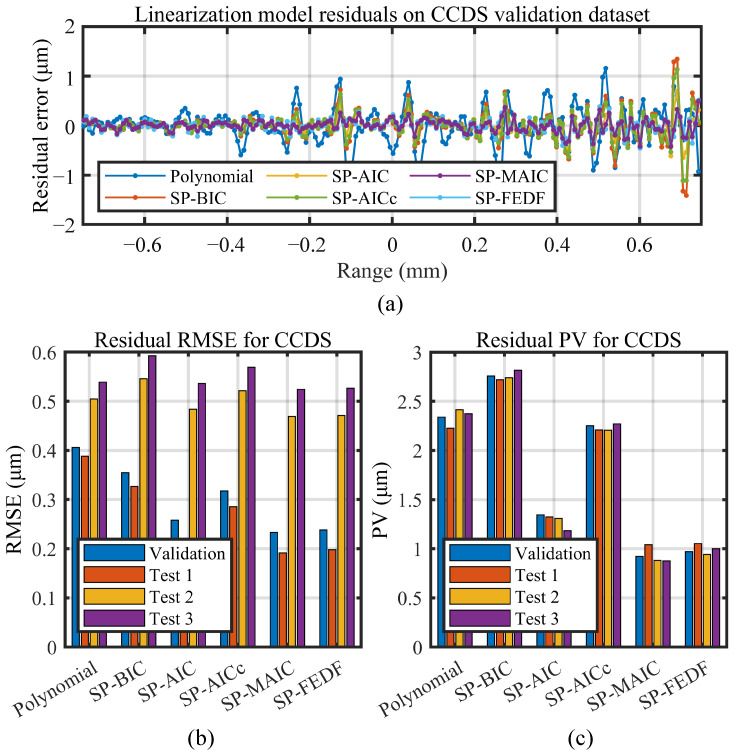
Linearization model performance on CCDS. (**a**) Validated nonlinearity residual error. (**b**) RMSE comparison of nonlinearity residuals for different models. (**c**) PV comparison of nonlinearity residuals for different models.

The residual error of all the selected models on the validation dataset along the ±0.75 mm valid sensing range are shown in Figure 11a. They are calculated via the deviation between the predicted value from each model and the validation data points. The spline models are generally more centralized along the mean axis of the data than the polynomial model. Figure 11b,c compares the residual nonlinearity RMSE and PV of each linearization method on the validation dataset and the three groups of test datasets. It is easy to see that for all the test datasets, the SP-MAIC had the least residual RMSE and PV compared to the SP-BIC, SP-AIC, SP-AICc, and SP-FEDF. This demonstrates the capability of the proposed smoothing spline linearization method with the MAIC criterion in balancing between underfitting and overfitting the nonlinearity data.

### 5.2. Linearization Results on the LTDS

**Figure 12:**
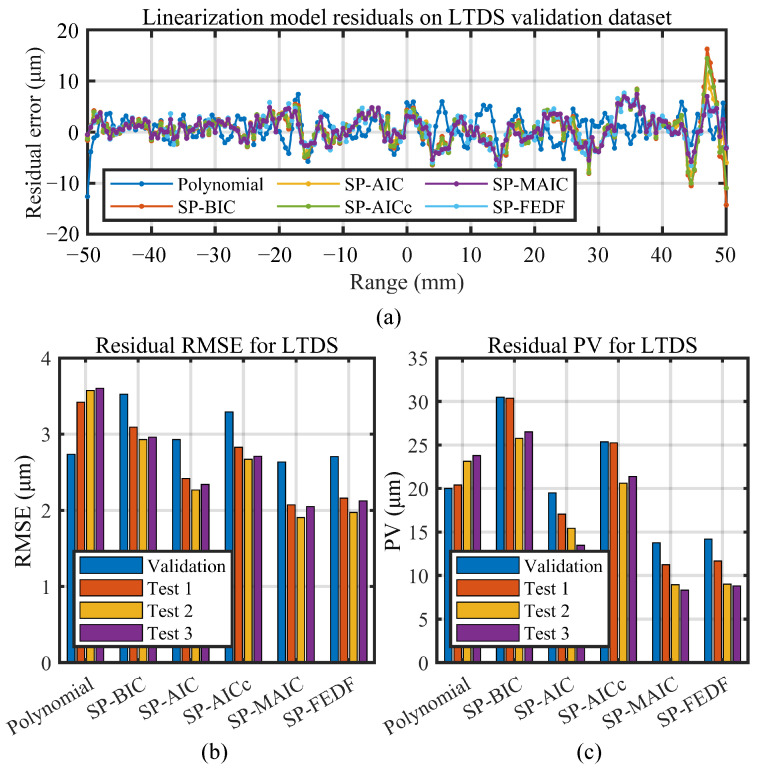
Linearization model performance on LTDS. (**a**) Validated nonlinearity residual error. (**b**) RMSE comparison of nonlinearity residuals for different models. (**c**) PV comparison of nonlinearity residuals for different models.

Similar to the data for CCDS, Table 3 and Figure 12 gives the testing results for LTDS along its ±50 mm measuring range. The SP-MAIC also performs the best among all the linearization methods. The testing results agree with those for the CCDS. The consistency of the results demonstrates the outstanding versatility of the SP-MAIC in accommodating sensors with distinct working principles and measuring ranges.

The linearization results are presented in detail in Figure 11 and Figure 12, Table 2 and Table 3. Notably, the two sensors both achieved sub-micron- to micron-level RMSE despite the significant difference in measuring range. The CCDS achieved a nonlinearity RMSE of 0.19 µm to 0.59 µm and a nonlinearity PV of 0.87 µm to 2.82 µm within its 1.5 mm measuring range, while the LTDS achieved a nonlinearity RMSE of 1.90 µm to 3.60 µm and a nonlinearity PV of 8.31 µm to 30.48 µm within its 100 mm measuring range. This results from the balanced oscillation of their nonlinearity residuals around a stable mean line value in Figure 11a and Figure 12a, where the 30 µm residual amplitude of the LTDS and the 3 µm residual amplitude of the CCDS are proportionally related to their micron- and sub-micron-level RMSE value.

From the linearization results across the three groups of repeated tests for the CCDS in Figure 11b,c, it can be observed that the RMSE of the latter two tests for the CCDS is higher than that of the first test, while their PV values are similar. This indicates that moderate change in nonlinearity residuals exist in the latter two test datasets, which could be attributed to the drift of the sensor system. However, for LTDS in Figure 12b,c, the linearization results on the three test datasets are similar. Despite this difference, the relative rank of the results for the linearization methods are the same for each test dataset, demonstrating the repeatability and stability of the proposed information-driven method for sensor linearization.

In Table 2 and Table 3, the nonlinearity error of the SP-AICc is higher than that of the SP-AIC. This contradicts common beliefs as the AICc usually perform better than AIC. The reason is that the AICc adds a higher penalization term to the model. For data with large noise, the AICc usually perform better. However, for the displacement sensors with small noise, the AICc has overpenalized the model, which leads to a worse performance. This is also the reason for proposing the MAIC criterion.

Additionally, it is noteworthy that the difference in linearization results between polynomial linearization, smoothing spline linearization, and the proposed non-smoothing spline linearization method with different information criterions in the experiments is not as distinguishable as in the simulation. From the bar charts in Figure 11 and Figure 12, the polynomial and the SP-FEDF perform better than the SP-BIC and SP-AICc. This is because the nonlinearity roughness of the two sensors are not as significant as the undulation adopted in Section 4.1 , which were enlarged for the purpose of distinction. From Figure 4a,b, the residual errors of the polynomial decrease significantly as local undulation becomes smaller and could potentially approximate the spline linearization results in principle. The phenomenon further reveals the superiority of SP-MAIC over the SP-BIC, the SP-AIC, and the SP-AICc in high-precision sensor linearization applications, which makes the main reason for proposing the fourth SP-MAIC. Moreover, for sensors with rougher nonlinearities, the robustness of SP-MAIC against large undulation is even more advantageous in achieving better linearization results.

### 5.3. Comparison of the Information Criterions for the Proposed Linearization Method

From the experimental data in Section 5.1 and Section 5.2, it can be concluded that the SP-MAIC achieved better results than the standard polynomial and non-smoothing spline models and also outperforms the information-driven linearization models with the other three criterions. This suggests that the SP-MAIC is a preferred choice against polynomials as well as the other three smoothing spline methods for sensor linearization. Among the parameter selection criterions for the smoothing spline model, the order of the EDF selected is SP-BIC < SP-AICc < SP-AIC < SP-MAIC, which complies with the metrics formulated in Section 2 where the rank of the penalty term is BIC > AICc > AIC > MAIC. In Figure 11 and Figure 12, the SP-BIC, SP-AIC, and SP-AICc perform worse than that selected via the non-smoothing cubic spline (SP-FEDF) because the BIC, AIC, and AICc criterion had overpenalized the model for the low noise sensors. Meanwhile, the fourth SP-MAIC performs even better than the cubic spline model through alleviating the influence of random noise in fitting. To make a direct comparison from the mean nonlinearity values after linearization via the polynomial model and SP-MAIC in Table 2 and Table 3, the proposed SP-MAIC had reduced the residual nonlinearity error by 60.1% for the chromatic confocal displacement sensor and 58% for the laser triangulation displacement sensor compared to the standard best-fit polynomial model. After conducting linearization, the remaining errors are evenly distributed around the zero axis in Figure 11a and Figure 12a. This suggests that the systematic errors that follow identifiable patterns are effectively eliminated, leaving random errors as the principal sources of the remaining errors.

## 6. Conclusions

To sum up, a novel information-driven smoothing spline linearization method integrated with three standard and one new information criterion had been proposed in this paper for linearization of high-precision sensors, among which the SP-MAIC constructed from the new MAIC criterion achieved the best linearization performance. Verification experiments had been conducted to compare the proposed method with the standard polynomial model and the non-smoothing spline model. The test results on two different kinds of self-developed sensors verified that the SP-MAIC demonstrated consistent best performance over other models and reduced the nonlinearities of the CCDS and the LTDS to ±0.0311% F.S. and ±0.0047% F.S., respectively. The residual errors were significantly decreased by over 50% compared to that by the best-fit polynomial model. Intrinsically, this work exploited the possible application of statistical methods like information criterions in improving traditional engineering applications, such as displacement sensor linearization, and could be further extended to the linearization and compensation of other physical systems. By combining smoothing spline with information criterions, this paper provides a generic and highly adaptable linearization method with the capability of managing intricate nonlinearities and noise, which could find broader applications in linearizing various complex and noisy systems.

While this paper focuses on the novel construction of smoothing spline linearization models via penalized information criterions for displacement sensors, the possible noise–range ratio of the sensor as to where the proposed metric performs best and how they can be adapted to other physical systems are also important topics that would be exploited in future work. For example, the validation experiments in this paper had been preliminarily conducted for noncontact opto-electronic sensors. For other types of sensors such as contact-type displacement sensors, additional influential factors, such as the geometry of the sensor tip and the characteristics of the target for nonlinearity measurements, are not addressed in this paper but are essential for determining the measurement nonlinearity.

After being linearized via the proposed method, the remaining residual errors are mainly composed of random errors such as electronic noise and thermal drift. In-depth work on controlling and reducing such random errors to further promote the accuracy of sensors is of equal importance and will be investigated in the following research. Building on the idea of compensating for systematic errors and eliminating random errors, further research could be conducted on compressing measurement noise and drift via digital filtering approaches. Additionally, the primary focus of this paper has remained on improving the linearization accuracy in practical conditions with noise. Further studies could evaluate and improve its computational efficiency to facilitate on-chip implementations.

## Figures and Tables

**Table 1:** Typical literature related to sensor linearization.

Reference	Model	Targeted Sensor	Summary of Contents
[19]	PL with periodic term	Laser interferometer	Developed a system to correct sub-fringe periodic nonlinearity using a capacitive sensor, achieving picometer-level repeatability.
[20]	Linear regression	Scale of motion axes	Utilized standard height artefacts to calibrate motion axes’ scale via linear regression, achieving ±0.043% F.S. for a coherence scanning interferometer.
[21]	PL	Inductive displacement sensor	Developed a laser interferometer for linearizing a 13 µm range sensor via third-order PL, attaining a theoretical nonlinearity of ±0.012% F.S..
[23]	Not applicable	Capacitive displacement sensor	Devised a Fabry–Perot interferometer to calibrate nanometer sensors up to a 300 µm range, with picometer resolution and nanometer uncertainty.
[28]	PL	Laser displacement sensor	Adopted a third-order PL for linearizing two non-contiguous intervals, reducing nonlinearity by up to 70% compared to linear interpolation.
[31]	PWL	General on-chip smart sensors	Implemented PWL for hardware linearization of smart sensors, focusing on cost-efficiency and memory optimization.
[32]	PWL	General types of sensors	Enhanced PWL models with alterable divisions for simplicity and high efficiency, achieving more than a 30% error reduction.
[33]	PWL	J-type and K-type thermocouple	Employed the included angle method for optimized PWL model construction, obtaining a nonlinearity of ±0.12% F.S. for a J-type thermocouple and ±0.41% F.S. for a K-type thermocouple.
[34]	PWL	General types of sensors	Constructed a PWL model that limits the maximum approximation error while retaining computational efficiency for microcontroller applications.
[35]	ANN	General types of sensors	Applied ANN in sensor linearization. Reduced nonlinearity of resistor sensors from ±3.33% F.S. to ±0.42% F.S..
[29,36]	PL and ANN	K-type thermocouple and thermistor	Applied both PL and ANN in linearizing thermal sensors. Achieved ±0.001% F.S. nonlinearity for a thermocouple and ±0.065% F.S. for a thermistor.
[37]	ANN	Thermistor	Showcased a 1-5-1 shallow ANN outperforming the hardware linearization method in linearization range and accuracy.
[39,40]	Bezier curve	Potentiometer displacement sensor	Employed Bezier curve modeling for sensor linearization, improving the position error from 14.2 mm to 4.2 mm.
[41]	S-spline	General on-chip smart sensors	Developed a recursive S-spline for on-chip linearization, achieving ±0.005% F.S. for an S-type thermocouple and ±0.175% F.S. for an NTC thermistor.
[42]	S-spline	Laser displacement sensor	Employed S-spline for contour reconstruction, reducing the measurement error from 282.91 µm to 252.55 µm in measuring an API thread.

**Table 2:** Residual nonlinearity error of linearization methods on CCDS (Unit: µm).

Model	Polynomial	Proposed Information-Driven Smoothing Spline				Cubic Spline (SP-FEDF)
		SP-BIC	SP-AIC	SP-AICc	SP-MAIC	
EDF	40	59	94	68	143	200
Validation	2.337605	2.755046	1.343160	2.249387	0.922966	0.967733
Test 1	2.225379	2.718632	1.321536	2.208768	1.038585	1.050816
Test 2	2.415095	2.738590	1.308218	2.204588	0.880576	0.942672
Test 3	2.373813	2.813443	1.182333	2.269050	0.875963	0.999185
Mean Nlt. (F.S.)	±0.0779%	±0.0919%	±0.0424%	±0.0742%	±0.0311%	±0.0333%

**Table 3:** Residual nonlinearity error of linearization methods on LTDS (Unit: µm).

Model	Polynomial	Proposed Information-Driven Smoothing Spline				Cubic Spline (SP-FEDF)
		SP-BIC	SP-AIC	SP-AICc	SP-MAIC	
EDF	38	53	75	59	138	200
Validation	20.008912	30.479826	19.482484	25.349988	13.764306	14.194509
Test 1	20.393406	30.371380	17.061518	25.258444	11.234457	11.685411
Test 2	23.133073	25.753753	15.416428	20.621292	8.939692	9.006504
Test 3	23.800611	26.495056	13.468149	21.371787	8.319668	8.794299
Mean Nlt. (F.S.)	±0.0112%	±0.0138%	±0.0077%	±0.0112%	±0.0047%	±0.0049%

## Data Availability

Data are contained within the article.
